# Deciphering Positive Cytomegalovirus Immunoglobulin M Test Results in Immunocompetent Adults Hospitalized With Illness Suspicious for Acute Cytomegalovirus Infection: A Multihospital Study

**DOI:** 10.1093/ofid/ofaf144

**Published:** 2025-03-10

**Authors:** Shrestha Samuel, Samantha Thomas, Louise Asleson, Anny Nguyen, Jeffery L Meier, Rima El-Herte

**Affiliations:** School of Medicine, Creighton University, Omaha, Nebraska, USA; School of Medicine, Creighton University, Omaha, Nebraska, USA; School of Medicine, Creighton University, Omaha, Nebraska, USA; School of Medicine, Creighton University, Omaha, Nebraska, USA; Iowa City Veterans Affairs Health Care System, Iowa City, Iowa, USA; Division of Infectious Diseases, Department of Internal Medicine, University of Iowa Carver College of Medicine, Iowa City, Iowa, USA; Division of Infectious Diseases, Department of Medicine, Creighton University School of Medicine and CHI Health, Omaha, Nebraska, USA

**Keywords:** hemophagocytic lymphohistiocytosis, CMV diagnosis, CMV disease immunocompetent, CMV IgG avidity, CMV IgM

## Abstract

**Background:**

Previously healthy adults hospitalized with an acute undifferentiated illness who test positive for cytomegalovirus (CMV) immunoglobulin M (IgM) in their serum may have a primary CMV infection, CMV reactivation/reinfection, or a false-positive result. We aimed to understand how clinicians interpret and incorporate positive CMV IgM test results into their diagnostic and management decisions.

**Methods:**

This was a retrospective case series study of 13 previously healthy, immunocompetent adults hospitalized with an acute illness in a 12-hospital system from 1 January 2017 to 1 January 2020, who tested positive for CMV IgM within 3 days of hospitalization. Twelve of 13 had CMV immunoglobulin G (IgG).

**Results:**

Among these 13 adults (median age, 36 years), elevated liver enzymes (100%), fever (85%), hepatosplenomegaly (54%), and headache (38%) were common. Lymphocytosis was observed in 5 patients, reactive lymphocytes in 3, and 1 patient died from hemophagocytic lymphohistiocytosis. Dual positivity for CMV and Epstein-Barr virus (EBV) IgM was frequent, yet only 1 patient was tested for both CMV and EBV DNA in blood or for CMV IgG avidity index, which indicated a primary CMV infection. Of the 6 patients with CMV DNA in blood, 4 received anti-CMV treatment. Uncertainty regarding CMV's role in the illness was common, and final assessments varied even among cases with similar clinical presentations and serologic patterns.

**Conclusions:**

Interpreting positive CMV IgM results in immunocompetent adults hospitalized with acute illness is challenging and ambiguous due to test limitations and confounders. Supplemental CMV IgG avidity testing can help determine whether primary CMV infection caused the illness, thereby refining the diagnosis and potentially influencing clinical decision-making.

Human cytomegalovirus (CMV) is a ubiquitous human-restricted herpesvirus that establishes lifelong infection. Both primary (acute) or a nonprimary (reactivation or reinfection) infection can cause debilitating or life-threatening disease in persons with compromised or immature cellular immune systems [1–5].

Most primary CMV infections in immunocompetent persons go unrecognized due to minimal or absent symptoms. In a small subset, primary CMV infection can present as heterophile-negative mononucleosis or prolonged fever with mild hepatitis [6, 7]. Severe illness or tissue-invasive disease is rare, but when it occurs, the clinical manifestations range widely depending on the affected organ system. The nonspecific nature of CMV-related symptoms and signs challenges even experienced clinicians, potentially delaying the diagnosis and increasing resource utilization.

Serological testing for CMV immunoglobulin M and G (IgM and IgG, respectively) is commonly used in immunocompetent persons to evaluate the possibility of whether CMV infection is contributing to the acute illness [2, 8, 9]. CMV IgG is usually present at illness onset, making serial CMV IgG testing impractical for demonstrating seroconversion from negative to positive, which would confirm primary CMV infection. CMV IgM is generally considered a marker of a primary (acute) CMV infection, because it appears during the acute illness and disappears within approximately 3 months. However, the time to disappearance of CMV IgM partly depends on the commercial IgM immunoassay type used [10]. Importantly, CMV IgM is an imperfect marker, as a positive result can also indicate CMV reactivation/reinfection or a false-positive result [8–10].

When both CMV IgM and IgG are present, the CMV IgG avidity test can help distinguish between acute/recent and past CMV infection [8, 9, 11–15]. A low CMV IgG avidity index combined with a positive IgM indicates a primary CMV infection, whereas a high IgG avidity index indicates the CMV infection occurred more than 3–4 months prior [12]. While the test is widely used to assess maternal CMV infection during pregnancy [8, 11–15], its availability and use vary by country [16]. In the United States (US), it is commercially available but is not US Food and Drug Administration (FDA) cleared, and test results often take several days to return in most hospitals.

Because healthy persons with latent CMV do not typically have levels of CMV DNA in whole blood or plasma, the presence of CMV DNAemia indicates active CMV infection. While CMV DNAemia supports the diagnosis of primary CMV infection [8, 17], it is not definitive proof, as it can also result from CMV reactivation in immunocompetent persons experiencing severe illness (eg, severe sepsis, prolonged critical illness, and extensive burns) [18–20]. Tissue biopsy evidence of invasive CMV disease in the setting of primary CMV infection in immunocompetent persons is rare.

There is a dearth of information about how clinicians interpret and incorporate positive CMV IgM test results in the evaluation and management of hospitalized patients with an acute undifferentiated illness. To explore this aspect of clinical practice, we carried out a systematic investigation of 13 immunocompetent adults hospitalized in a 12-hospital healthcare system for an acute illness and a positive CMV IgM test. Our findings highlight the clinical spectrum of illness and underscore the wide variation in diagnostic test utilization, clinical management, and the clinical team's final assessment of CMV's role in the illness.

## METHODS

### Study Design and Ethical Statement

This is a retrospective case series study conducted within a US Midwest healthcare system comprising 12 hospitals in 2 states from 1 January 2017 through 1 January 2020. The local institutional review board granted a full waiver of consent and Health Insurance Portability and Accountability Act (HIPAA) authorization for this research.

### Study Population

The study enrolled previously healthy adult males and females hospitalized for an acute illness, with a positive CMV IgM test obtained within the first 3 days of admission. An electronic search of medical records from 104 370 adult patients hospitalized for at least 24 hours—excluding those admitted for rehabilitation or observation—identified individuals with a positive CMV IgM result. Manual review of the records of these patients by a board-certified infectious diseases subspecialist determined that 13 patients met the study criteria. Patients were excluded if they had an immunocompromising disease; were receiving an immunosuppressing medication; or had an active malignancy, inflammatory bowel disease, or autoimmune disease. Active inflammatory bowel disease and immunosuppression are known to trigger CMV reactivation, while autoimmune disease increases likelihood of false-positive CMV IgM tests. Among the 13 eligible patients, 1 had a prior history of alcohol use disorder, which was not a contributing factor in their illness, and another had Langerhans histiocytosis in childhood, which had been in remission for >25 years.

### CMV and EBV Laboratory Tests

The Bio-Rad BioPlex platform was used to measure CMV IgM and IgG and EBV serologies. The FDA-cleared BioPlex multiplexed flow immunoassays, applied to detect CMV IgM and EBV viral capsid antigen (VCA) IgM, utilize beads coated with lysates of CMV and an *Escherichia coli*–derived recombinant fusion protein, EBV VCA p18 (40 kD), respectively. Results for CMV IgM, CMV IgG, and EBV VCA IgM were reported as positive or negative, without quantitative values. CMV avidity IgG testing and polymerase chain reaction (PCR) testing for CMV or EBV DNA in plasma were performed at ARUP Laboratories (Salt Lake City, Utah).

### Data Collection

Data elements of interest were manually collected from the patients’ electronic medical records and entered in a secure database. Captured data elements include age; sex at birth; past medical history; and symptoms, signs, and physical examination findings at hospital presentation. Laboratory test results from the first week of hospitalization, including a complete blood cell count with differential, a complete metabolic panel (with liver function tests), and infectious disease tests, were recorded. Findings of invasive procedures, imaging, and pathology studies performed during the hospitalization were also documented. Deaths, use of anti-CMV drugs, and *International Classification of Diseases, 10th Revision* (*ICD-10*) diagnosis codes for CMV disease (B25.9), CMV mononucleosis (B27.10), herpesviral infection unspecified (B00.9), and unspecified viral infection (B34.9) were also recorded. An infectious diseases subspecialist reviewed the electronic medical records to cross-check the entered data and determine the clinical team's final assessment as to whether CMV was a possible, probable, or definite cause of the illness.

### Statistical Analysis

Summary statistics were applied to describe categorical (percentages) and continuous (mean, median, and range) variables.

## RESULTS

We collected data on 13 immunocompetent adults with no prior significant health conditions who were hospitalized with an acute illness in a multihospital healthcare system in the US Midwest. CMV was included in the differential diagnosis, and a positive CMV IgM test was obtained within 3 days of hospitalization (Table 1). The median age of patients was 36 (range, 25–82 years), and 38% were female (Table 2).

**Table 1.** Summary of Immunocompetent Adults Hospitalized for Acute Illness With Positive Cytomegalovirus Immunoglobulin M Test

Patient # Age/Sex	Symptoms and Objective Findings	CMV Blood Tests				CMV Diagnosis	CMV Rx	Outcome, *ICD* Code: CMV/Virus
		IgM	IgG	IgG Avidity	DNA			
Pt 1 35/M	Fever, cough, dyspnea, sweats, jaundice No pharyngitis or palpable LN CT of abdomen and pelvis with HSM; LEE; pancytopenia; increased liver enzymes; AKI EBV VCA IgG^+^ IgM^−^; EBNA^+^; EA^−^; Toxo Ab ND HLH diagnosis; death before HLH treatment	+	+	ND	+ 202 copies/mL	Possible CMV-induced HLH, unclear if primary CMV or reactivation	GCV	Death by multiorgan failure *ICD* cd: Ø
Pt 2 82/M	Fever, cough, dyspnea, joint pain, anorexia Encephalopathy, oligoarticular arthritis, edema in extremities No pharyngitis or palpable LN WBC and differential normal; increased liver enzymes; EBV and hepatitis A/B/C virus serology panels negative; Toxo Ab ND CXR and US of abdomen unremarkable	+	+	ND	ND	Possible CMV disease, primary CMV infection	None	Survived *ICD* cd: Ø
Pt 3 34/M	Anorexia, jaundice, weakness Prior history of alcohol use disorder Jaundice, no pharyngitis or palpable LN WBC 13K, N 70%, L 16% Increased liver enzymes and bilirubinemia; EBV and hepatitis A/B/C virus serology panels negative; HSV-1 IgG^+^, HSV-2 IgG^−^; Toxo Ab ND US abdomen and MRCP: HSM and intrahepatic duct dilation Liver biopsy with cellular and canalicular cholestasis; no steatosis, hepatitis, or malignancy Negative stain for CMV	+	+	ND	+ <200 IU/mL	Possible CMV disease, possible primary CMV infection	VGCV	Survived *ICD* cd: Ø
Pt 4 32/F	Fevers, sore throat, chest pain, cough, dyspnea Histiocytosis as child Pharyngitis, HSM, and abdominal tenderness WBC 13K, L 55%, +2 reactive lymphocytes, increased liver enzymes EBV VCA IgG^+^ IgM^+^; EA Ab^−^; EBNA^+^; EBV DNA^+^ qual; hepatitis A/B/C virus serology panel negative; Toxo Ab ND CT chest with GGO, mediastinal lymph nodes and US abdomen with HSM	+	+	ND	ND	Possible CMV or EBV disease, primary CMV or reactivation not addressed	None	Survived *ICD* cd: CMV disease
Pt 5 53/M	Fever and headaches No pharyngitis or palpable LN WBC 7.9K with L 34%, +1 reactive lymphocytes; thrombocytopenia, increased liver enzymes, and acute renal failure Syphilis and *Coxiella* Ab^−^; parvovirus IgG^+^ IgM^−^; EBV and hepatitis A/B/C virus serology panels negative; Toxo Ab ND CSF normal CT of chest, abdomen, and pelvis with HSM	+	–	ND	+ 1460 IU/mL	Probable CMV disease, probable primary CMV infection	VGCV	Survived *ICD* cd: CMV disease
Pt 6 27/F	Fever, headaches, cough, dyspnea, abdominal pain, nausea, vomiting, anorexia, and skin rash HSM, skin rash, and abdominal tenderness; no pharyngitis or palpable LN WBC and differential normal, increased liver enzymes and bilirubin EBV VCA IgM^+^ IgG^+^; EA Ab^−^; EBNA^+^; heterophile Ab^−^; WNV IgM^−^, IgG^−^; hepatitis A/B/C virus serology panel negative; Toxo Ab ND CT of chest, abdomen, and pelvis with HSM	+	+	ND	ND	Possible CMV disease, probable primary CMV infection	None	Survived *ICD* cd: CMV disease
Pt 7 44/F	Fever, dyspnea, abdominal pain, nausea, vomiting, anorexia HSM; no pharyngitis or palpable LN WBC 17K, L 68%; increased liver enzymes and ferritin EBV VCA IgG^+^ IgM^+^; EA Ab^−^; EBNA^+^; *Histoplasma* and *Cryptococcus* serum Ag^−^; hepatitis A/B/C virus serology panel negative; Toxo Ab ND Bone marrow with increased T cells; CT chest with lung nodules, GGO, pleural effusion, and HSM	+	+	+ low	+ 13 700 IU/mL	Probable CMV disease from reactivation. Postdischarge, primary CMV	VGCV	Survived *ICD* cd: Ø
Pt 8 53/M	Fever, headaches, abdominal pain, joint pain, sweats Abdominal tenderness; no pharyngitis or palpable LN WBC and differential normal; AKI; increased liver enzymes EBV serology panel negative; Toxo Ab ND CT of chest, abdomen, and pelvis with GGO	+	+	ND	–	No CMV disease, primary CMV or reactivation not addressed	None	Survived *ICD* cd: Ø
Pt 9 46/F	Fever, headaches, dyspnea, abdominal pain, nausea Normal physical exam; increased liver enzymes and LDH WBC 25K, L 72% EBV VCA IgG^+^ IgM^+^; EBNA^+^; EA Ab^−^; EBV DNA^−^; hepatitis A/B/C virus and Toxo serologies ND *Histoplasma* Ag serum and urine negative CT of the chest, abdomen, and pelvis: mediastinal LN, small pleural effusion, gallbladder stones; bone marrow biopsy with T-cell large granular lymphocytes; cholecystectomy with chronic cholecystitis on pathology	+	+	ND	+ 41 000 copies/mL	CMV disease from primary CMV infection	None	Survived *ICD* cd: CMV disease
Pt 10 57/F	Fever, headaches, cough, dyspnea, abdominal pain, nausea, vomiting, diarrhea, anorexia No pharyngitis, palpable LN, or HSM WBC 12K, L 51%; increased liver enzymes EBV VCA IgG^+^ IgM^+^; EA Ab^+^; EBNA^+^; EBV DNA^+^ qual; hepatitis A/B/C serology panel negative; Toxo Ab ND	+	+	ND	ND	Disease from primary CMV or EBV	None	Survived *ICD* cd: Other viral agent
Pt 11 36/M	Seizure with altered mental status Encephalopathy; no pharyngitis or palpable LN WBC normal, L 3%; thrombocytopenia; renal failure; increased liver enzymes MRI increased long T2 signal mesial temporal lobes bilaterally, right greater than left; CSF normal CSF encephalitis/meningitis PCR panels twice negative (includes HSV, CMV, and HHV-6) EBV and hepatitis A/B/C serology panel negative; Toxo, syphilis, WNV serologies negative; HSV-2 IgG^+^; EBV serologies ND	+	+	ND	ND	Not CMV disease, CMV reactivation. Treated for possible HSV encephalitis	None	Survived *ICD* cd: Unspecified herpesvirus infection
Pt 12 29/M	Fever, cough, sweats; wife had mono syndrome 1–2 wk prior No pharyngitis, no palpable LN or HSM WBC 13K, L 69%, reactive lymphocytes; increased liver enzymes and LDH EBV VCA IgG^+^ IgM^+^; EA Ab^−^; EBNA^+^; HSV-1/2 IgM^+^ IgG^−^ No hepatitis A/B/C panel or Toxo Ab CT chest: old granulomatous disease	+	+	ND	+ 805 copies/mL	Drug fever (NSAID), insignificant CMV reactivation	None	Survived *ICD* cd: Ø
Pt 13 34/M	Fever, jaundice, cough, dyspnea, skin rash, abdominal pain, nausea, sore throat, LN neck, sweats Skin rash, jaundice, abdominal tenderness, submandibular LN WBC 17K with N 72%, L 13%; increased liver enzymes and bilirubin EBV VCA IgG^+^ IgM^+^; EA Ab^−^; EBNA^+^; negative IM screen, *Leptospira* IgM, RPR, and cryptococcal serum Ag; CSF unremarkable; Toxo Ab ND MRCP with HSM	+	+	ND	ND	Possible CMV disease, possible primary CMV infection	None	Survived *ICD* cd: Ø

Abbreviations: −, negative; +, positive; Ø, without CMV or viral diagnosis; Ab, antibody; Ag, antigen; AKI, acute kidney injury; ALT, alanine aminotransferase; AST, aspartate aminotransferase; CMV, cytomegalovirus; CSF, cerebrospinal fluid; CT, computed tomography; CXR, chest X-ray; EA Ab, antibody against Epstein-Barr virus early antigen; EBNA, antibody against Epstein-Barr virus nuclear antigen; EBV, Epstein-Barr virus; F, female; GCV, ganciclovir; GGO, ground glass opacification in lung; HHV-6, human herpesvirus type 6; HLH, hemophagocytic lymphohistiocytosis; HSM, hepatosplenomegaly; HSV, herpes simplex virus; *ICD* cd, *International Classification of Diseases, 10th Revision* diagnosis code; IM, infectious mononucleosis; L, leukocytes; LDH, lactate dehydrogenase; LEE, lower extremity edema; LN, lymph node; M, male; MRCP, magnetic resonance cholangiopancreatography; MRI, magnetic resonance imaging; N, neutrophils; ND, not done; NSAID, nonsteroidal anti-inflammatory drug; qual, qualitative; RPR, rapid plasma reagin; Rx, prescription; Toxo, *Toxoplasma*; VGCV, valganciclovir; VCA, viral capsid antigen; US, ultrasound; WBC, white blood cell count; WNV, West Nile virus.

**Table 2.** Clinical and Laboratory Features

Characteristic	No. (%)
Sex	
Female	5 (38)
Male	8 (62)
Age, y	
25–35	6 (46)
36–47	3 (23)
48–57	3 (23)
≥58	1 (8)
Median (range)	36 (25–82)
Fever	11 (85)
Headaches	5 (38)
Lymphadenopathy	1 (8)
Pharyngitis	1 (8)
Hepatosplenomegaly	7 (54)
Pulmonary infiltrates	2 (16)
Increased liver enzymes	13 (100)
Jaundice	2 (16)
HLH	1 (8)
Normal WBC count	6 (46)
Lymphocytosis	5 (38)
Reactive lymphocytes	3 (23)
Neutrophilia	2 (16)
CMV IgG positive	12 (92)
CMV IgG avidity index	1 (8)
Plasma CMV DNA testing	7 (54)
CMV DNA detected	6 (46)
EBV serology obtained	9 (70)
Positive EBV VCA IgM	7 (54)
Positive EBV VCA IgG	8 (62)
Positive EBNA antibody	8 (62)
Plasma EBV DNA testing	3 (23)
EBV DNA detected	2 (15)
Hepatitis virus testing	7 (54)

Abbreviations: CMV, cytomegalovirus; EBNA, Epstein-Barr virus nuclear antigen; EBV, Epstein-Barr virus; HLH, hemophagocytic lymphohistiocytosis; IgG, immunoglobulin G; IgM, immunoglobulin M; VCA, viral capsid antigen; WBC, white blood cell.

### Clinical Features

Eleven patients had fever (85%), 5 had headache (38%), 1 had pharyngitis, and 1 had submandibular lymphadenopathy. Hepatomegaly and splenomegaly were present in 7 patients (54%), while 2 had lung infiltrates. All patients exhibited biochemical evidence of hepatitis. Among the 2 patients with visible jaundice, 1 had hemophagocytic lymphohistiocytosis (HLH), and the other underwent a fine-needle liver biopsy, which revealed cellular and canalicular cholestasis and negative immunostaining for CMV. Five patients had lymphocytosis, with reactive lymphocytes noted in 3. Six patients had a normal white cell count, 2 had neutrophilia, and the patient with HLH had pancytopenia and lymphopenia.

### Laboratory Testing

Twelve of 13 patients had no detectable CMV IgG, but only 1 underwent CMV IgG avidity testing. Among the 7 patients who underwent PCR testing for CMV DNA in blood, 6 (86%) had detectable CMV DNAemia, and 4 received anti-CMV treatment with ganciclovir or valganciclovir. EBV serologies were obtained in 9 patients, with 7 showing dual positivity for CMV IgM and EBV viral capsid protein (VCA) IgM. Half of the patients underwent serologic testing for hepatitis viruses, while testing for other infectious causes varied by case.

### Outcomes and Clinician Assessments

The only death occurred in a 35-year-old man (patient 1) who was hospitalized with fever, cough, jaundice, hepatosplenomegaly, and pancytopenia, which were attributed to HLH leading to multiorgan failure. The patient had detectable CMV IgM, CMV IgG, and DNAemia (202 copies/mL) and was treated with IV ganciclovir. The clinical team's diagnosis of possible CMV-induced HLH was not captured in the *ICD-10* codes for this patient. All 12 surviving patients showed clinical improvement by discharge.

Clinicians identified CMV as a possible cause of the illness in 6 cases, probable cause in 2, and likely cause in 2. However, only 4 cases had *ICD-10* diagnosis codes reflecting CMV's role in the illness. Primary CMV infection was considered possible in 5 cases, probable in 2, and likely in 2. In 3 patients, CMV was ruled out as the cause of illness, although in 2 of these cases, no alternative etiology was identified.

### Subset Analysis of Variability in Clinician Decision-Making

To illustrate differences in clinician decision-making, we examined 5 patients who shared fever, hepatitis, peripheral lymphocytosis, and dual positivity for CMV IgM and EBV VCA IgM (patients 4, 7, 9, 10, and 12). All had CMV IgG, EBV VCA IgG, and antibodies to EBV nuclear antigen (EBNA). Two had reactive lymphocytes (patients 4 and 12), and 2 had hepatosplenomegaly (patients 4 and 7).

Three patients (patients 7, 9, and 12) underwent CMV PCR testing on blood, all of whom had CMV DNAemia (range, 805–41 000 copies/mL). Patient 9 also underwent qualitative PCR testing for EBV DNA in plasma, which was undetectable. Following an extensive workup—including computed tomography of chest, abdomen, and pelvis, a bone marrow biopsy, and cholecystectomy—this patient was diagnosed with likely CMV disease resulting from a primary infection and clinically improved without antiviral treatment.

Patient 7 had hepatosplenomegaly, pulmonary infiltrates, and peripheral lymphocytosis. The clinician's final assessment was probable CMV disease due to viral reactivation. However, a CMV IgG avidity test, performed before discharge, later showed a very low value of CMV IgG avidity index, indicating that a primary CMV infection was the likely cause. Patient 12, who also had reactive lymphocytes, was diagnosed with drug fever and inconsequential CMV reactivation.

The 2 patients who did not undergo CMV PCR testing (patients 4 and 10) tested positive for EBV DNA in plasma. Their illness was attributed to either EBV or CMV, and they did not receive antiviral treatment. None of the 5 patients were tested for acute toxoplasmosis, which can present with fever, liver enzyme elevations, hepatosplenomegaly, lymphocytosis, and pulmonary infiltrates [21]. Two patients (patients 4 and 7) had fever, hepatitis, hepatosplenomegaly, lymphocytosis, and lung infiltrates.

## DISCUSSION

CMV IgM is commonly regarded as a marker of primary (acute) CMV infection, often in the context of illnesses resembling heterophile-negative mono syndrome or a febrile episode with elevated liver enzymes [6, 7], in the absence of confounders that might complicate the diagnosis. However, a positive CMV IgM test should be interpreted with caution, particularly in cases with atypical features. In such cases, the result may indicate CMV reactivation/reinfection or a false-positive test. Multiple studies on maternal CMV infections indicate that most positive CMV IgM tests in early to mid-pregnancy do not result from primary CMV infection [5]. Additionally, commercial CMV IgM immunoassays vary in specificity and exhibit differing levels of IgM cross-reactivity in the context of a primary EBV infection [10]. Severe illness or inflammatory conditions can also trigger CMV reactivation, diminishing the reliability of a positive CMV IgM test in identifying primary CMV infection in such settings. Clinical guidelines advise against the use of CMV IgM tests to diagnose acute CMV illness in transplant patients [22] or patients with AIDS [23], because the test is more likely to yield misleading results in immunocompromised populations.

Acute CMV infection is appropriately considered in the differential diagnosis of the illnesses affecting the hospitalized patients in this case series. Among the 13 cases, common findings included liver enzyme elevation (100%), fever (85%), hepatosplenomegaly (54%), and headache (38%). Peripheral lymphocytosis was observed in 5 patients (38%), 3 had reactive lymphocytes, and 1 developed and died from HLH.

Our study highlights substantial variability in how clinicians evaluate these patients and assess CMV's role in their illness, even among cases having a similar clinical presentation and serologic test result patterns. In most instances, clinicians were uncertain whether the positive CMV IgM test indicated primary infection, reactivation, or a false-positive result. Only 40% of patients deemed to have possible, probable, or likely CMV disease received an *ICD-10* diagnosis code for clinically significant CMV infection. Anti-CMV drugs were administered to 31% of all patients and 66% of the those with CMV DNAemia. However, we could not determine whether antiviral treatment significantly impacted clinical outcome.

The finding that 54% of the 13 study patients had dual positivity for CMV IgM and EBV VCA IgM adds complexity to the clinical decision-making. Our findings align with those of Jhaveri et al, who report that dual positivity for CMV IgM and EBV IgM is common [24]. In our study, all patients with a positive EBV IgM also had antibody to EBNA, a serological pattern suggesting EBV reactivation or a false-positive EBV VCA IgM test, as EBNA antibodies are typically absent during primary EBV infection [25]. Additionally, primary CMV infection has been reported to induce IgM antibodies that cross-react with EBV VCA [26–29].

Among the 6 patients with dual CMV IgM and EBV VCA IgM positivity, only 1 underwent PCR testing for both CMV and EBV DNA in plasma. In this case, the presence of CMV DNA (41 000 copies/mL) and absence of EBV DNA supported the clinician's impression of CMV disease likely due to primary CMV infection. Quantifying both CMV and EBV DNA in plasma may help identify the causative virus. However, this approach has limitations, as severe primary infection with 1 virus could potentially trigger reactivation of the other virus.

CMV IgG avidity testing has become a valuable tool for distinguishing primary CMV infection from CMV reactivation/reinfection or false-positive CMV IgM results. While it has been most extensively studied and incorporated into obstetric guidelines [5, 8, 9, 14], its utility in other immunocompetent populations is increasingly recognized [13, 29–32]. It has also been used to assess prevalence of recent CMV infection in children and women of childbearing age in the US [33, 34].

In this case series, the only patient who underwent CMV IgG avidity testing had low CMV IgG avidity index, supporting a diagnosis of primary CMV infection. This result helped clarify a dual CMV IgM and EBV VCA IgM positivity in a 41-year-old woman who presented with fever, hepatosplenomegaly, pneumonitis, liver enzyme elevation, peripheral lymphocytosis with reactive lymphocytes, and CMV DNAemia (13 000 IU/mL). In the other suspected acute CMV cases where both CMV IgM and IgG were present, CMV IgG avidity testing could have helped determine whether the positive CMV IgM results indicated a primary CMV infection or instead reflected CMV reactivation/reinfection or false-positive result. Further research is needed to assess whether incorporating CMV IgG avidity testing into routine practice improves clinical decision-making, resource utilization, patient satisfaction, and clinical outcomes.

We conclude that the use of positive CMV IgM test results in clinical decision-making is challenging due to test limitations and case-specific confounders. Relying solely on a positive CMV IgM test to diagnose acute CMV infection is problematic, and supplemental testing is necessary to support this diagnosis. While the broad differential diagnosis of acute undifferentiated illness guides the initial workup, CMV IgG avidity testing can help determine whether primary CMV infection is the cause when both CMV IgM and IgG are present.

This study has limitations inherent to its retrospective case series design, including lack of a control group and potential for selection bias and incomplete patient data. Additionally, our findings may not be generalizable to other healthcare systems or patient populations. Variability in the performance of commercial tests used to measure CMV IgM and CMV IgG avidity potentially limits the applicability of this study.
